# A Young Patient with Leg Weakness and Hypokalemia—Case Report

**DOI:** 10.5041/RMMJ.10330

**Published:** 2018-04-19

**Authors:** Elias Andrawus, Irit Hochberg, Zaher S. Azzam

**Affiliations:** 1 Department of Internal Medicine B, Rambam Health Care Campus, Haifa, Israel; 2 Institute of Endocrinology, Diabetes and Metabolism, Rambam Health Care Campus, Haifa, Israel; 3 The Ruth & Bruce Rappaport Faculty of Medicine, Technion, Israel Institute of Technology, Haifa, Israel

**Keywords:** Conn’s disease, hypokalemia, primary hyperaldosteronism

## Abstract

A 20-year-old female patient was admitted to hospital because of bilateral leg weakness. Laboratory investigation showed metabolic alkalosis and severe hypokalemia. Differential diagnosis included mineralocorticoid or apparent mineralocorticoid excess diseases, with a high aldosterone-to-renin ratio (ARR) after correcting hypokalemia. After confirmatory tests, imaging studies revealed a unilateral adrenocortical adenoma consistent with Conn’s disease. Surgery was curative.

## CASE PRESENTATION

A 20-year-old female patient was admitted to the emergency department because of bilateral leg weakness. At presentation, she had severe hypokalemia of 1.4 mM, and an electrocardiogram (ECG) showed U waves. She was admitted to the Cardiac Intensive Care Unit for intravenous potassium replacement. Following laboratory correction of potassium levels to 4.2 mM, she was transferred to our internal medicine department. The patient recalled experiencing a gradual bilateral leg weakness, sensation of numbness, and occasional dull pain for over two weeks. Serial blood pressure measurements showed elevated values around 160/110 mmHg. The patient is known to have asymptomatic protein S deficiency. Notably, she denied other concomitant complaints, including: fever, weight loss, gastrointestinal or genito-urologic complaints, rash, drug abuse, treatment with over-the-counter medications or oral contraceptives, alcohol consumption, suicidal thoughts, travel to foreign countries, and special contact with animals.

Her family medical history included a mother with protein S deficiency. In addition, her grandfather died in the early 1970s at the age of 30 years from “kidney disease and resistant high blood pressure.” In September 1969 he was admitted to another hospital due to “essential hypertension”: systolic blood pressure was 180–200 mmHg and diastolic was 120–150 mmHg, creatinine clearance was low and serum urea was high, serum electrolytes were reported within the normal range, and renal angiography was negative for vascular disorder; he was treated with methyldopa, hydralazine, and furosemide.

Returning to our patient, on admission to our department she was hemodynamically stable, her blood pressure was 148/98 mmHg, pulse 90/min regular, temperature 37°C per os, and she was euvolemic. Otherwise, the physical examination was unrevealing.

### Laboratory Tests

On admission, laboratory tests revealed the following:

- Arterial blood gases: pH, 7.45; HCO_3_^−^, 37 mmol/L; pCO_2_, 55 mmHg; l-lactate, 1.2 mmol/L; osmolarity, 288 mmol/L.
- Blood chemistry: Na, 137 mmol/L; K, 2.3 mmol/L; Mg, 1.2 mmol/L; creatinine, 0.28 mg%; blood urea nitrogen (BUN), 5 mg%; uric acid (UA), 1.8 mg%; albumin, 3.3 g%; aspartate aminotransferase (AST), 180 U/L; creatinine phosphokinase (CPK), ~7,220 U/L; other electrolytes and liver function tests were within normal ranges.
- Hematology: Hemoglobin, 11.6 g%; mean corpuscular volume (MCV), 77 fL; leukocytes, 12.4×10^9^/L; normal white blood cell differential; normal platelet count and international normalized ratio (INR).
- Endocrinology: Thyroid stimulating hormone (TSH) and free thyroxine (FT4) within normal ranges.
- Viral serology: negative serology (ELISA) for human immunodeficiency virus (HIV).

### Differential Diagnosis

The patient was admitted with severe hypokalemia as an emergency, necessitating treatment before initiation of investigation.

During her hospital stay, the patient was treated with high-dose oral and intravenous potassium supplements. Her diet was enriched with potassium; her serum K ranged between 2.5 mM and 3.2 mM. Magnesium levels were corrected early during the first days.

In this patient, the first step in investigation has aimed to establish the differential diagnosis (DD) of hypokalemia and metabolic alkalosis.

The DD of hypokalemia includes three main categories. First, decreased potassium intake is not relevant because no clinical signs of malnutrition and no other laboratory nutritional deficiencies were observed, and hypokalemia was persistent despite continuous potassium replacement. Second, potassium redistribution into cells can also cause hypokalemia, mainly due to hormones, drugs, and anabolic states (e.g. insulin, β-agonists, granulocyte-colony stimulating factor [G-CSF] analogues, vitamin B12 supplements); however, all of these cases were excluded by the history and laboratory tests. Two other specific diseases, familial hypokalemic periodic paralysis and thyrotoxic periodic paralysis, are also not relevant, because, in the former, hypokalemia should be medically corrected by supplements, and, in the latter, thyroid battery tests should show hyperthyroidism.

The third category, which is relevant to this case, includes potassium renal loss via mineralocorticoid or apparent mineralocorticoid excess diseases.

The DD of metabolic alkalosis also led to mineralocorticoid or apparent mineralocorticoid excess diseases, because the patient had no vomiting or other gastrointestinal volume loss; urinary screening for toxic substances and for diuretics was negative, and the patient denied chewing licorice.

Therefore, following the initial investigation that integrated a thorough history, physical examination, and preliminary laboratory test, the most likely DD were as follows:

1. Primary hyperaldosteronism (PA), including: bilateral adrenal hyperplasia, Conn’s disease (adrenocortical adenoma), and type 1 familial hyper-aldosteronism, glucocorticoid remediable aldosteronism (GRA)
2. Renal artery stenosis (fibromuscular dysplasia)
3. Other familial hyperaldosteronism (other than type 1, GRA)
4. Syndrome of apparent mineralocorticoid excess
5. Cushing syndrome
6. Renin-secreting tumor
7. Liddle syndrome

### Laboratory and Imaging during Hospitalization

Because of the high clinical and laboratory suspicion for PA, a screening test for the aldosterone-to-renin ratio (ARR) was performed. Laboratory units were reported in mU/L for direct renin concentration (DRC) and pmol/L for plasma aldosterone concentration (PAC).

The result of the initial screening test was negative; DRC was 15 mU/L and PAC was 625 pmol/L, hence the calculated ARR was 41, whereas for a positive screening test it should be >90. Notably, serum potassium was 3.1 mM during the exam, which may cause a false negative result.

Later, the test was repeated following the correction of serum potassium levels with escalating doses given both orally and intravenously; a PA conformatory test using saline infusion was also performed. The laboratory results are summarized in Table 1.

**Table 1:** Laboratory Results of Saline Infusion Conformatory Test.

Time/Laboratory Results	Plasma Potassium (mM)	DRC (mU/L)	PAC (pmol/L)	Plasma Cortisol (nmol/L)
Before saline infusion	3.4	Immeasurable	902	299
After saline infusion	3.4	Immeasurable	873	203
Aldosterone-to-renin ratio		∞	∞	

The PAC may be reported in different units; ng/dL or pmol/L. Renin may be measured directly (DRC), or activity may be measured (plasma renin activity [PRA]). Different cut-off values are relevant to each measurement.^1^

While awaiting laboratory results, Doppler ultrasound of the kidneys was completed with no evidence of renal artery stenosis, and three consecutive 24-hour urine collections for free cortisol were within the normal range.

In addition to potassium replacement therapy, the patient was treated with one oral agent for hypertension, lercanidipine (a non-dihydropyridine calcium channel blocker) up to 20 mg/day, with a systolic blood pressure target of 130 mmHg.

### Confirming Diagnosis

**Figure 1:**
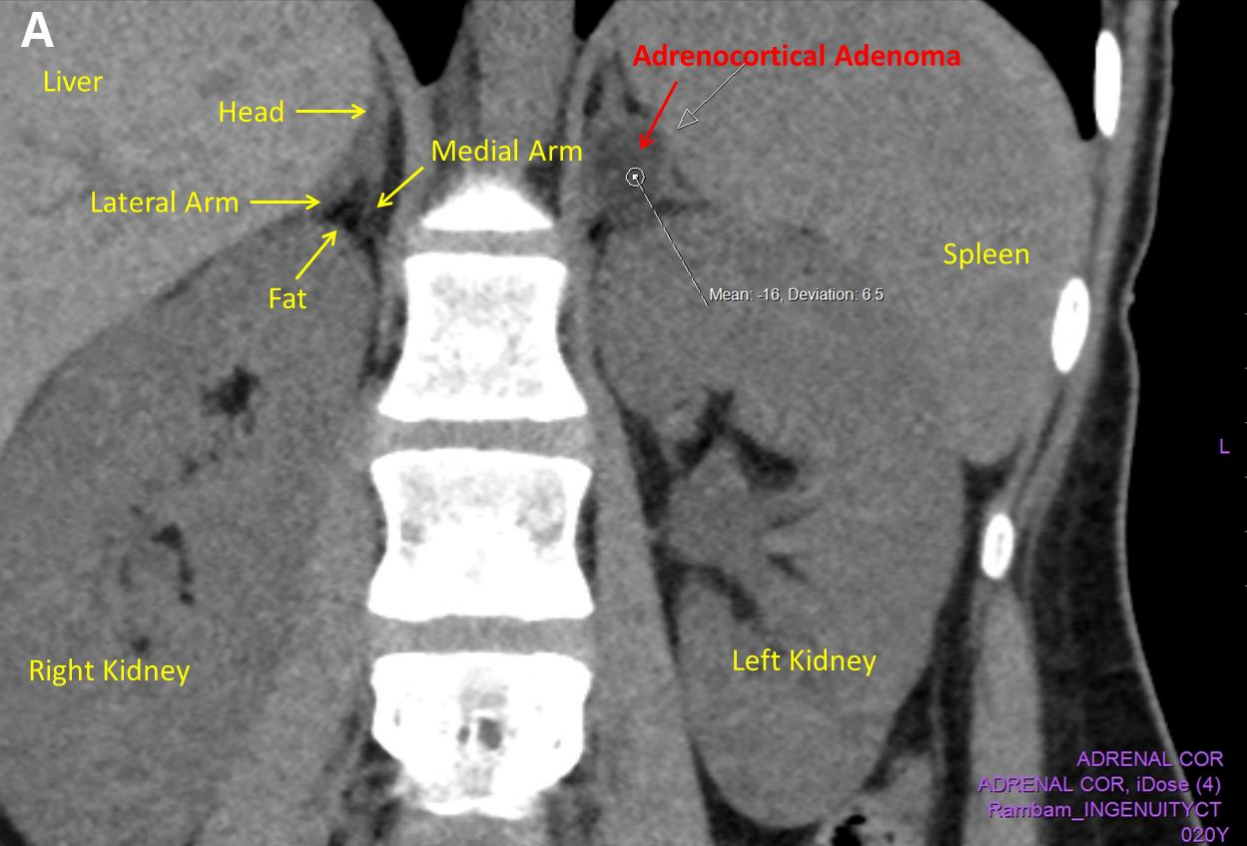

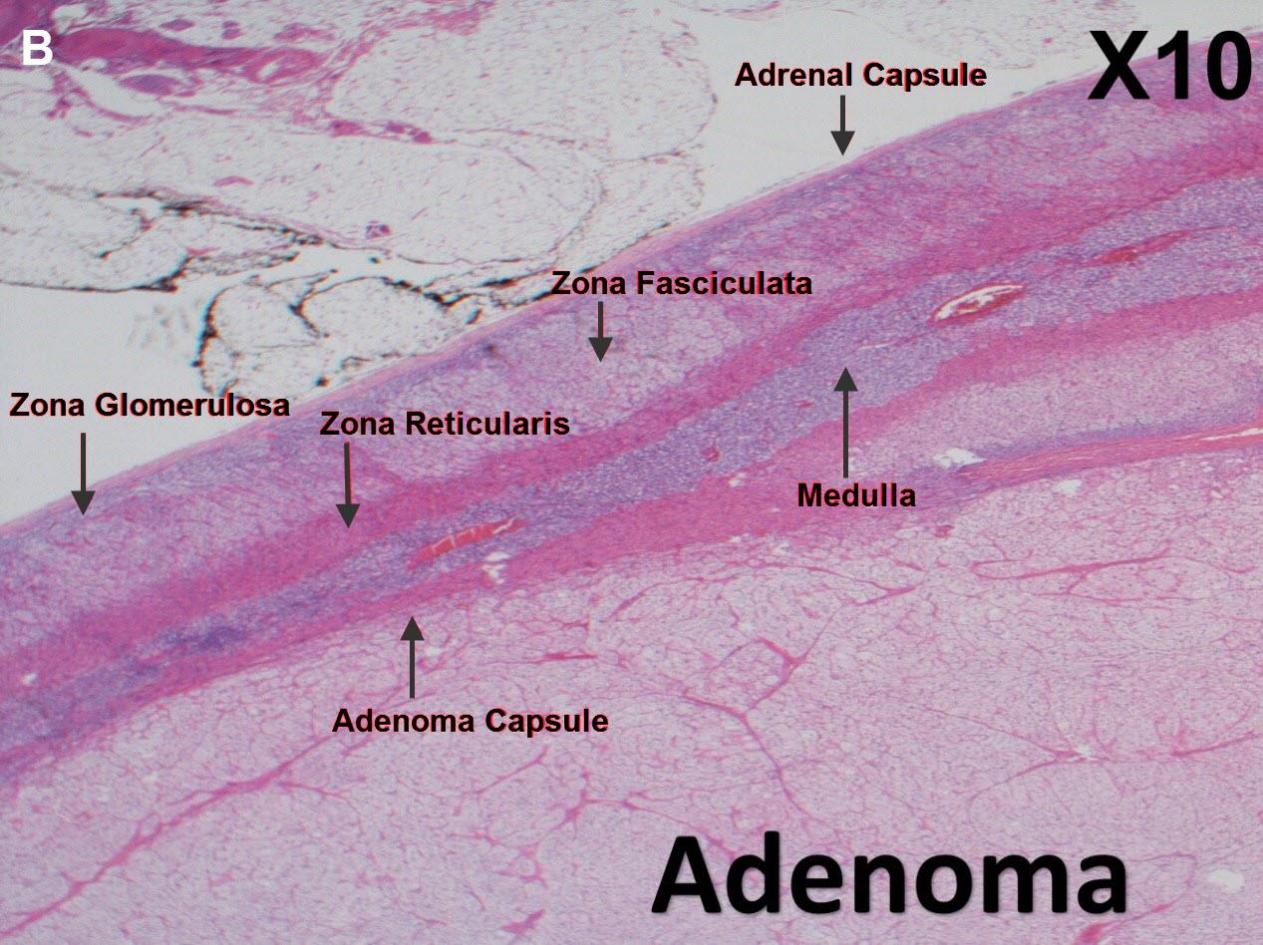
CT Imaging and Histopathology of Left Adrenocortical Adenoma **A:** A coronal section of unenhanced adrenal CT showing a left adrenocortical adenoma. **B:** Higher magnification of adenoma tissue (H&E stain, ×10 magnification).

After the laboratory results confirmed PA, an unenhanced computerized tomography (CT) of adrenals was completed, which showed an adenoma (1.6 cm × 1.8 cm × 2 cm) in the left adrenal; the Hounsfield unit density was negative (–16), which is consistent with adrenocortical adenoma (Figure 1A).

Because the patient was young (<40 years) with an adenoma and biochemical confirmation of PA, she was referred directly to an endocrine surgeon^1^,^2^ and underwent a left laparoscopic adrenalectomy.

### Histopathologic Results

Gross pathology showed a mass, 1.6 cm wide, within the adrenal tissue, and a microscopic view demonstrated cells with neither mitosis, nor atypia, nor necrosis. The margins were free (Figure 1B).

No special histologic stain for aldosterone was available. Moreover, there is no correlation between the microscopic appearance of the adenoma and either of the three “adrenal zonas.”

### Patient Follow-up

After surgery, the patient remained hypokalemic for 4 days and required high doses of potassium supplements. After 7 days she was readmitted to the internal medicine ward, where serum potassium laboratory follow-up showed slight hyperkalemia (4.8–5.4 mM) due to a transient hyporeninemic state.^3^

The patient was discharged to ambulatory endocrinologist follow-up. Blood pressure and serum potassium were followed, and both were normal for 3 months without need for medications or supplements.

One year later, the medical team of the internal department contacted the patient by phone; she signifies feeling well, with no specific complaints. Referring to her primary care physician, serum potassium in July 2017 was reported as 4.4 mM.

## DISCUSSION

Primary hyperaldosteronism (PA) is a common cause of secondary resistant hypertension.^1^–^3^ Within PA, Conn’s disease and bilateral adrenal hyperplasia are most common.^2^

Notably, PA is considered underdiagnosed; prospective studies have estimated that PA constituted ~10% of mild-to-moderate essential hypertension,^1^,^3^–^5^ with the main morbidity and mortality of cardiovascular complications.^1^,^6^,^7^

Hypokalemia is an insensitive screening tool for PA; sensitivity ranges between 9% and 37%, and only half of patients with Conn’s disease have hypokalemia.^1^

The ARR is considered the most reliable test for PA screening,^1^,^5^ although, like other laboratory tests, it has false positive results (e.g. due to beta-adrenergic blockers usage, renal failure, and aging) and false negative results (e.g. due to hypokalemia, K-wasting drug usage, and pregnancy).^1^

This case indicates that the test should be repeated if there is high clinical and laboratory suspicion after listing the appropriate DD, as we had a negative screening result at first (maybe due to hypokalemia or a technical laboratory mistake).^1^

Beside the somatic (intratumor) potassium channel (SCJN5, GERK mutation), several other mutations have been discovered in PA and other familial hyperaldosteronism variants. Their clinical relevance is still unclear; it is noteworthy that there are consensus guidelines incorporating genetic testing as part of the diagnosis.^1^,^8^–^10^

Lastly, even after our patient had recovered from her disease, several questions are still unanswered, such as: Is there a relationship between the patient’s case and her grandfather’s case? Is there a germline or somatic (intratumor) mutation? Should genetic sequencing be completed, and, if so, will it have clinical relevance?

In summary, we describe a patient with severe hypokalemia and hypertension; a diagnosis of primary hyperaldosteronism was made. The patient was treated medically and surgically with complete recovery.

## Abbreviations

ARR: aldosterone-to-renin ratio
DD: differential diagnosis
DRC: direct renin concentration
GRA: glucocorticoid remediable aldosteronism
PA: primary hyperaldosteronism
PAC: plasma aldosterone concentration.
